# A systematic review and meta-analysis of diagnostic delay in pulmonary embolism

**DOI:** 10.1080/13814788.2022.2086232

**Published:** 2022-06-22

**Authors:** R. van Maanen, E. M. Trinks-Roerdink, F. H. Rutten, G. J. Geersing

**Affiliations:** Julius Center for Health Sciences and Primary Care, University Medical Center Utrecht, Utrecht University, Utrecht, The Netherlands

**Keywords:** Pulmonary embolism, venous thromboembolism, delay, diagnosis, systematic review, meta-analysis

## Abstract

**Background:**

Diagnostic delay in patients with pulmonary embolism (PE) is typical, yet the proportion of patients with PE that experienced delay and for how many days is less well described, nor are determinants for such delay.

**Objectives:**

This study aimed to assess the prevalence and extent of delay in diagnosing PE.

**Methods:**

A systematic literature search was performed to identify articles reporting delays in diagnosing PE. The primary outcome was mean delay (in days) or a percentage of patients with diagnostic delay (defined as PE diagnosis more than seven days after symptom onset). The secondary outcome was determinants of delay. Random-effect meta-analyses were applied to calculate a pooled estimate for mean delay and to explore heterogeneity in subgroups.

**Results:**

The literature search yielded 10,933 studies, of which 24 were included in the final analysis. The pooled estimate of the mean diagnostic delay based on 12 studies was 6.3 days (95% prediction interval 2.5 to 15.8). The percentage of patients having more than seven days of delay varied between 18% and 38%. All studies assessing the determinants of coughing (*n* = 3), chronic lung disease (*n* = 6) and heart failure (*n* = 8) found a positive association with diagnostic delay. Similarly, all studies assessing recent surgery (*n* = 7) and hypotension (*n* = 6), as well as most studies assessing chest pain (*n* = 8), found a negative association with diagnostic delay of PE.

**Conclusion:**

Patients may have symptoms for almost one week before PE is diagnosed and in about a quarter of patients, the diagnostic delay is even longer.

Key messages

- In this systematic review and meta-analysis with an extensive scope of all existing relevant studies on delay in diagnosing pulmonary embolism (PE), the mean diagnostic delay was almost one week and in a quarter of patients the delay was even longer.
- This emphasises the importance of increasing awareness on PE and educating patients and physicians on how to recognise PE.

## Introduction

Pulmonary embolism (PE) is the most serious condition within the spectrum of venous thromboembolic (VTE) conditions, given its associated high mortality rate, as well as its related morbidity and frequent hospitalisation [1,2]. Prompt and early recognition of PE is thus paramount. Clinical prediction rules – such as the Wells criteria, Geneva rule or YEARS algorithm – can assist physicians in diagnosing PE in suspected patients [3–5]. However, these rules are useful only when the physician has a clinical suspicion of PE. It can be extremely challenging to diagnose PE on time because symptoms of PE can differ widely in severity, and are often non-specific [6,7]. In some patients ultimately diagnosed with PE, the suspicion either never arose or occurred only after multiple consultations. For example, the so-called ‘classical’ PE-triad of chest pain, dyspnoea, and haemoptysis occurs in less than 10% of patients [8].

Insight into the proportion of patients with PE that experienced delay and determinants associated with delay may help to increase awareness among physicians and patients, and thereby help to reduce diagnostic delay. This is especially meaningful for general practitioners (GPs) since patients with symptoms of PE often seek medical advice from their GP first. No previous study has systematically assessed the prevalence and extent of delay in diagnosing PE. Therefore, the purpose of this study was to systematically review the literature on studies reporting on delay in diagnosing PE. The primary objective was to assess the proportion of patients with PE that experienced diagnostic delay and the extent of this delay. A secondary objective was to identify determinants associated with a delayed diagnosis of PE.

## Methods

### Search strategy

On 31 August 2021, we performed a literature search in Medline and Embase databases without date limits or language restrictions. The key terms in the search consisted of ‘pulmonary embolism’ and synonyms, combined with ‘diagnostic delay’, ’time to diagnosis’, ‘misdiagnosis’ and alternative terms (See Online Appendix 1 for the full search syntax). Two reviewers (RvM and EMTR) screened the abstracts independently and selected original studies, describing any form of delay in the diagnostic management of PE. Subsequently, both reviewers independently selected full-text articles. In case of no consensus between these two researchers selecting a full-text article, a third researcher (GJG) was asked to screen the article in question, and a consensus was reached by discussion. We performed a cross-reference check for all included articles.

### Definitions and study selection

For this study, ‘diagnostic delay’ was defined as the time between the onset of symptoms (as reported by patients and described in the original publication) until confirmation of the diagnosis of PE. The primary objective was to quantify the presence of ‘diagnostic delay’, expressed as either a mean or median delay, or as a percentage of patients with diagnostic delay more than seven days. The secondary objective was to quantify determinants for such delay. Studies conducted in general practices, emergency departments and hospital wards were considered for this review. We excluded systematic reviews, case reports, and articles describing the outcome in a particular population, e.g. paediatric populations, only post-operative patients or pregnant women. Also, articles that only considered ‘logistic delay’, for example, the time between admission and confirmation of the diagnosis with imaging, were excluded from our review since our primary aim was to obtain a pooled point estimate of the total diagnostic delay. Finally, if there was no definition of delay mentioned or if we could not derive the definition of delay, the article was excluded.

### Risk of bias and applicability assessment

No validated risk of bias tool was available for observational cross-sectional studies when we performed this review. Therefore, two reviewers independently assessed the risk of bias with modified criteria based on the QUADAS-2 tool [9]. We scored the risk of bias as high, low or unclear, within the following three domains: selection of study population (to assess generalisability and selection bias), validity of diagnostic testing (to assess information bias) and assessment of delay (to assess recall and information bias). Moreover we scored the applicability of studies to primary care. Studies performed in general practice or studies in which the GP referred patients are considered very applicable to primary care. Studies in which a part of the included patients were referred by their GP are considered likely applicable to primary care. Studies in which patients were included from emergency departments are considered as possibly applicable. Studies in which patients were included from hospital wards are deemed not applicable to primary care. If it was unclear from which setting patients were included, we considered the applicability to primary care as unclear. See Online Appendix 2 for the modified risk of bias and applicability tool used, including further clarification of these domains.

### Data extraction and data analysis

The data were extracted using a standardised data extraction form. In addition to the primary objective to assess diagnostic delay of PE, we also collected data concerning our secondary objective, i.e. determinants for delay. Both determinants tested in univariable analysis and determinants tested in multivariable analysis were considered. We created an overview of clinically relevant determinants studied more than once and described whether a (significant) positive or negative association was found in the individual studies.

We performed a meta-analysis with studies that reported a mean delay since most studies reported a mean delay and not a median delay. Studies only reporting a median delay were excluded from this meta-analysis. We have sought contact with authors of studies only reporting a mean delay to obtain the median delay as well but unfortunately, we received no response. We log-transformed the data because we assumed that the mean delay of the individual studies was not normally distributed. Random-effects meta-analysis was applied to calculate a pooled estimate with a 95% confidence interval and prediction interval for the mean diagnostic delay (defined in days). The prediction interval represents the range of estimates for the mean delay that can be found in future studies with a similar study design and thus can be considered as a measure of heterogeneity across studies [10]. Next, we performed meta-analyses to explain the heterogeneity in the following subgroups: studies that included only patients in the emergency department, studies with a low risk of bias due to misclassification, studies with the same definition of delay (time from onset of symptoms to diagnosis) and studies with prospective and retrospective data collection. Statistical analyses were performed in R version 3.4.1.

## Results

**Figure 1.**
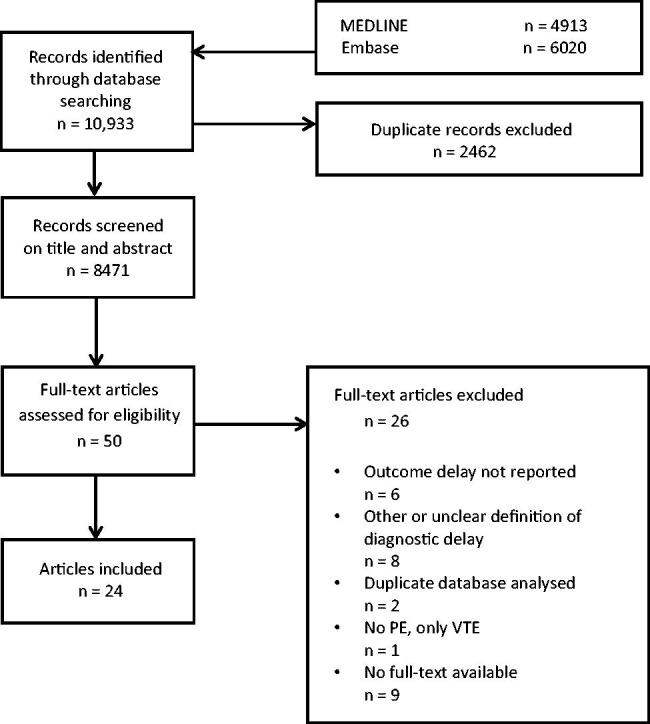
Flow-chart article selection.

The literature search yielded a total of 10,933 studies. After screening on title and abstract, we identified 50 articles, which we assessed for eligibility. Twenty-four articles met our in-and exclusion criteria [11–34]. For an overview of the literature search and article selection, see Figure 1. The 24 studies were published between 1998 and 2021. Data were collected retrospectively in 13 studies and collected prospectively in 11 studies. The included studies were performed in different settings, namely: primary care practices (*n* = 1), emergency departments (*n* = 7), hospital wards (*n* = 9) or combinations (*n* = 7). The characteristics of the included studies are presented in Table 1. The risk of bias regarding the domains of patient selection and valid diagnosis was assessed as ‘low’ in most studies. The risk of bias due to misclassification (assessment of delay) was assessed as ‘high’ in 10 studies, mostly because of retrospective data collection. Two studies were assessed as very applicable to primary care, five studies as likely applicable, five studies as possibly applicable, six studies as not applicable and for six studies the applicability to primary care was unclear. See Online Appendix 3 for the risk of bias and applicability assessment.

**Table 1.** Studies that assessed diagnostic delay in patients with pulmonary embolism.

Study *Name first author + year of publication*	*n* *Patients with PE*	Patient characteristics *Mean age + SD (years)*	Female (%)	Setting inclusion *Emergency department (ED), during hospital admission (HA), general practice (GP)*	Design of data collection	Definition delay *Moment of start counting delay – moment of stop counting delay*	Mean delay *Mean (days) + standard deviation*	Dela*y* > 7 days *Percentage of patients in this category*	Other categories of delay
Ageno 2008 [11]	542	59.8	57.4	HA + ED	Prospective	Symptoms – diagnosis			<5 days: 64% 5–10 days: 20% >10 days: 16%
Alonso-Martínez 2004 [12]	106	72 ± 11	46.2	HA	Prospective	Symptoms – hospital admission	10 ± 12		
Alonso-Martínez 2010 [23]	375	Median 75 IQR 15	49.6	HA	Prospective	Symptoms – diagnosis	Median 6 IQR 12		>6 days: 50% >14 days: 25% >21 days: 10%
Aranda 2021 [28]	150	61.2 ± 18	51.3	HA	Prospective	Symptoms – diagnosis		26%	
Aydoǧdu 2013 [29]	53	65 ± 17	54.7	ED	Prospective	Symptoms – diagnosis	6.8 ± 7.7	38%	>1 day: 93%
Berghaus 2011 [30]	248	64.2 ± 16.4	60.5	HA	Retrospective	Symptoms – diagnosis	2.5 ± 1.9		
Bulbul 2009 [31]	178	60.4 ± 16.8	53.9	HA + ED	Retrospective	Symptoms – diagnosis	9.3 ± 11.6		
Bulbul 2011 [32]	156	64.1 ± 15.9	62.2	ED	Prospective	Symptoms – diagnosis	7.93 ± 10.05		
Chan 2020 [33]	302	^a^	^b^	HA	Retrospective	Symptoms – diagnosis		24%	
den Exter 2013 [34]	849	52 ± 18/56 ± 18^c^	58.2^d^	HA + ED	Prospective	Symptoms – diagnosis		19%	
Elliott 2005 [13]	344	61.3 ± 16.4	57.3	HA	Retrospective	Symptoms – diagnosis	4.8 ± 20.2	17%	>25 days: 5%
Goyard 2018 [14]	514	Median 65 IQR28	51.2	HA	Prospective	Symptoms – diagnosis	Median 3 IQR 8	27%	>3 days: 47%
Hendriksen 2017 [15]	128	56 ± 15/62 ± 18^e^	53.1	GP	Retrospective	First GP contact – diagnosis		26%	
Ilvan 2015 [16]	100	58.31 ± 15.13	46	ED	Retrospective	Symptoms – diagnosis	11.9 ± 22.6	28%	
Jenab 2014 [17]	195	59.2 ± 17.1	42.1	ED	Prospective	Symptoms – presentation hospital	5.6 ± 7.9		<1 day: 31% <3 days: 57% >1 month: 1%
Jiménez Castro 2007 [18]	397	69	55.4	ED	Prospective	Symptoms – diagnosis	Median 7	18%	>25 days: 6%
Kayhan 2012 [19]	189	57.95 ± 16.36	55.0	HA	Retrospective	Symptoms – diagnosis		37%	
Menéndez 1998 [20]	102	64 range 21–88	54.9	HA + ED	Retrospective	Symptoms – diagnosis	Median 4 range 3–11		
Ozlem 2016 [21]	11	71.5 ± 7.9	72.7	ED	Retrospective	Symptoms – ED admission	10.6 range 3–30		
Ozsu 2011 [22]	408	62.12 ± 16.2	57.4	HA + ED	Retrospective	Symptoms – diagnosis	6.9 ± 8.5	28%	
Pasha 2014 [24]	113	56 ± 17	46.9	HA + ED	Retrospective	Symptoms – presentation hospital	5.7 ± 9.2	18%	>1 month: 4%
Rahimi-Rad 2013 [25]	88	54.46 ± 17.27^f^	43.6^g^	HA + ED	Prospective	Symptoms – treatment	3.05 ± 6.42		
Walen 2016 [26]	261	60.6 ± 16.9	47.9	ED	Retrospective	Symptoms – diagnosis	8.6 ± 25.5	24%	>1 month: 6%
Zycińska 2013 [27]	53			HA	Retrospective	Symptoms – diagnosis	5		

^a^115 patients (38.1%) <65 years, 152 patients (50.3%) 65–84 years and 35 patients (11.6%) ≥85 years.

^b^77 female patients (67.0%) <65 years, 100 female patients (65.8%) 65–84 years and 25 female patients (71.4%) ≥85 years.

^c^Complaints < 7 days: 52 ± 18, complaints >7 days: 56 ± 18 (suspected PE patients).

^d^Suspected female PE patients.

^e^56 ± 15 (no diagnostic delay) 62 ± 18 (diagnostic delay).

^f^Baseline characteristics of 353 patients with PE, PE/DVT or DVT.

^g^43.6% female patients in a group of 353 patients with DVT, PE and DVT + PE patients.

### Diagnostic delay

**Figure 2.**
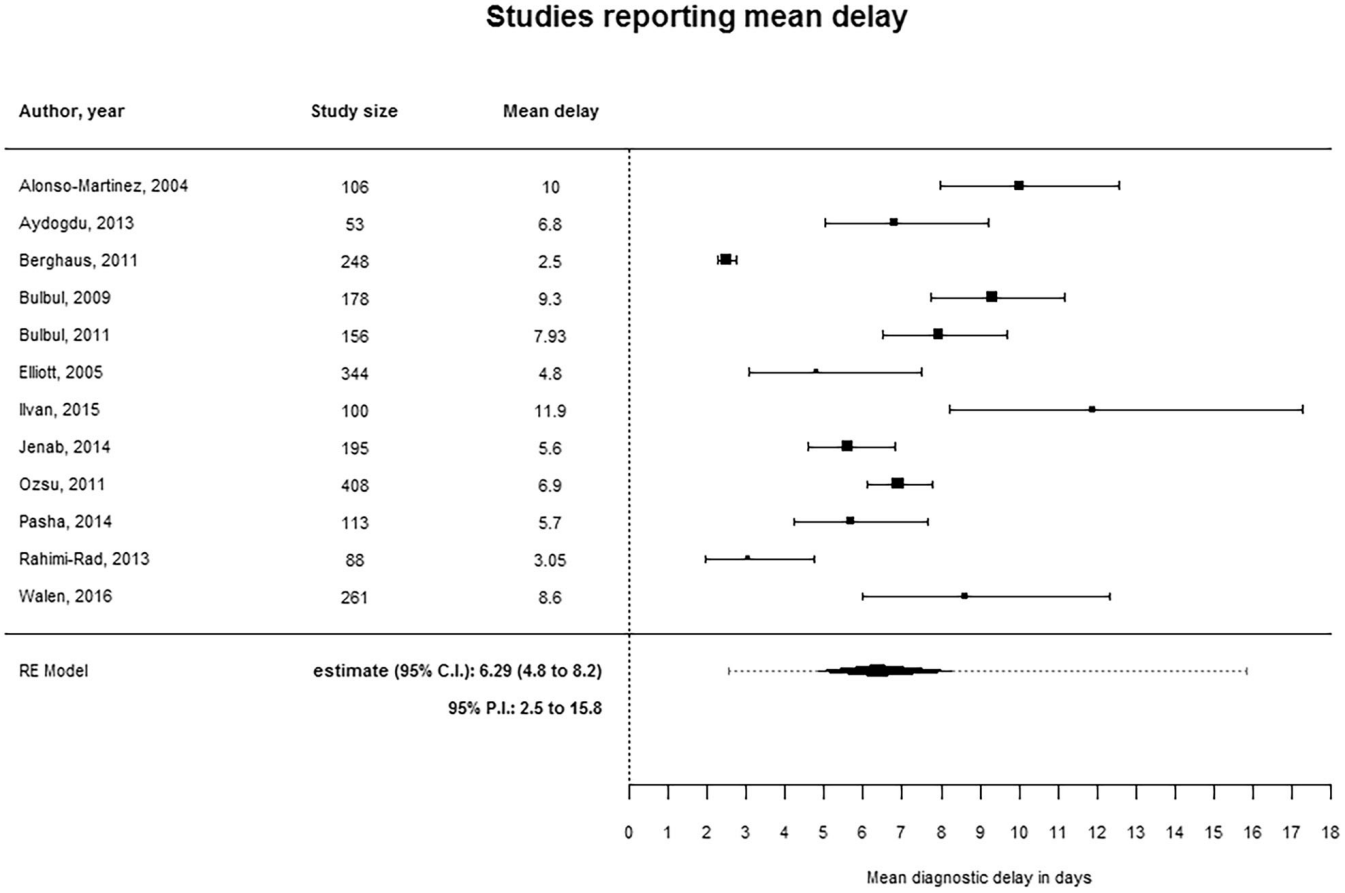
Meta-analysis of studies reporting mean delay.

In total, 12 studies presented a mean delay with standard deviation. Figure 2 shows the forest plot of all 12 studies reporting a mean delay in diagnosing PE. The reported mean delay ranged from 2.5 to 11.9 days. The pooled point estimate of the mean delay was 6.3 days (95% CI 4.8 to 8.2) with a wide prediction interval (95% PI 2.5 to 15.8 days). The mean delay in studies performed in emergency departments was 7.7 days (95% PI 4.6 to 12.8). In our further pre-defined subgroup analyses (i.e. analyses of only studies with a low risk of bias, with a uniform definition of delay, or only using either prospective or retrospective data collection) the prediction intervals remained wide, indicating residual and unexplained heterogeneity. Sixteen studies reported a percentage of patients with diagnostic delay. Thirteen of these fifteen studies categorised delay beyond seven days. More than seven days of delay varied between 18% and 38%. The primary outcomes are presented in Table 1.

### Determinants associated with delay

**Figure 3.**
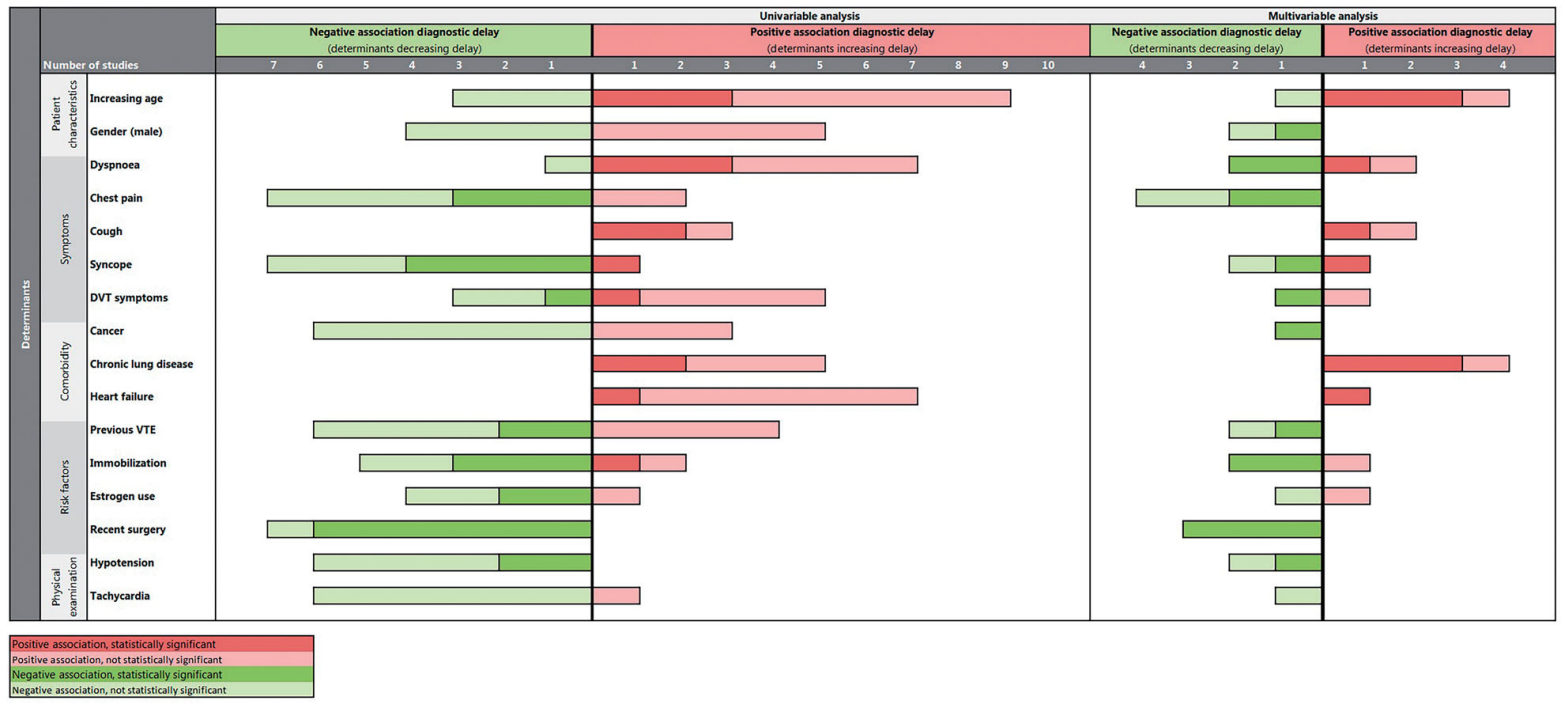
Determinants associated with diagnostic delay.

Fourteen studies assessed determinants potentially associated with diagnostic delay. Figure 3 summarises these determinants and the positive or negative association with diagnostic delay found in the individual studies (See Online Appendix 4 for the complete overview). For many of the explored determinants, findings were inconclusive and sometimes conflicting across different studies. Nevertheless, from a narrative synthesis, we identified several determinants positively and negatively associated with diagnostic delay based on univariable and/or multivariable analyses, albeit not all statistically significant (Figure 3, Online Appendix 4). First, all of the three studies analysing coughing symptoms, all of the six studies analysing chronic lung disease and all of the eight studies analysing heart failure found a positive association of these determinants with diagnostic delay. Second, all of the seven studies analysing recent surgery and all of the six studies analysing hypotension found a negative association of these determinant with diagnostic delay. Finally, seven out of nine studies analysing chest pain and six out of seven studies analysing tachycardia found a negative association with diagnostic delay.

## Discussion

### Main findings

This systematic review shows that delay in diagnosing PE is common, with a pooled point estimate of a mean diagnostic delay of almost one week, albeit with a wide prediction interval indicating considerable heterogeneity between studies. About a quarter of patients had more than seven days of delay. Existing data suggest that patients with chronic cardiopulmonary co-morbidity or symptoms of coughing are at greater risk for delay. Yet, these observations were made only out of narrative synthesis from the included studies as formal meta-regression on determinants for delay was considered in appropriate due to differences in determinant definition and analytical techniques used.

### Strengths and limitations

To the best of our knowledge, this is the first study to systematically describe the full scope and extent of delay in diagnosing PE. We performed a complete literature search without date or language restrictions and could provide an extensive scope of all existing relevant studies. Thereby, we were able to summarise the existing body of evidence on this important topic, hoping to provide some ‘base evidence’ for future studies embarking on this topic, allowing to compare findings from these new studies with the inferences found in our review. Furthermore, we pooled the mean delay using random-effect meta-analyses and explored heterogeneity. Some limitations, however, need to be taken into account. First, the mean diagnostic delay in days is probably not normally distributed, so providing a pooled estimate of the median delay would have been preferable. However, most studies only reported a mean delay with a standard deviation and therefore, we had to use the mean delay to calculate a pooled estimate. Second, in some of the included studies, delay was not clearly defined, necessitating us to use a proxy instead. The definition of delay also differed between the studies. Most of the included studies analysed the time from the onset of symptoms until the definitive confirmative diagnosis of PE. However, some studies reported the time from onset of symptoms until hospital admission, emergency department admission, or the start of treatment. For future diagnostic studies on PE, we would recommend reporting on diagnostic delay uniformly. We would suggest reporting the time between symptom onset (patient-reported) and confirmation of the PE diagnosis, and preferably also the time between symptom onset and the moment that the patient seeks medical attention to distinguish between patients and physicians delay. Third, the methodology of the included studies differed, for example, in determining the duration of diagnostic delay. In some studies, patients were interviewed after a confirmative diagnosis, which could introduce recall bias, which is difficult (or even impossible) to adjust for. Finally, probably as an overall consequence of these above-described limitations, the between-study heterogeneity was considerable. An essential cause of heterogeneity was that patients were included from different settings (hospital wards, emergency departments and primary care). In our review both studies categorised as very applicable to primary care, found a similar percentage of patients delay of more than seven days (24% and 26%). However, since both patient and physician delays and the clinical implications of delay will be largely dependent on the setting of inclusion, this should be considered when interpreting our results.

### Clinical implications

In our review, we focussed primarily on the prevalence and extent of diagnostic delay of PE. Although not the purpose of our study, we could hypothesise on possible explanations for the diagnostic delay of approximately a week. First and foremost, it might be that PE-symptoms are often not timely recognised by the physician and/or the patient. As mentioned before, symptoms of PE are often non-specific and can vary in severity. Consequently, it can be challenging to differentiate PE from alternative diagnoses, leading to a delay in the diagnostic process. This is supported by the fact that we found that delay seemed to occur more frequently in patients with comorbidities. Moreover, the decreasing prevalence of proven PE in suspected patients in diagnostic studies might suggest that physicians do think of PE quite often but still are struggling to correctly and timely identify PE in the right patients [35,36]. This emphasises the importance of increasing awareness of PE and educating physicians and patients on how to recognise PE, e.g. during (albeit not exclusively) events like World Thrombosis Day [37].

Second, another explanation for the diagnostic delay we found might be that PE is not an acute disease per se in *all* PE patients. With an average duration of symptoms almost a week before diagnosis, PE might rather be a subacute condition with slower onset of unfolding symptoms in a subset of patients, leading to a ‘delayed’, or perhaps better framed as a protracted and evolving, presentation. Should this be true, it could be that the delay in diagnosis *might* be associated with less negative clinical consequences in the patients with such a milder clinical trajectory. In that respect, it could well be that delay happens more often in patients with sub-segmental PE than in patients with lobular or more central PE’s. Both possible explanations could also be valid simultaneously. Yet, given that PE can also have profound (long-term) implications, more research is urgently needed to gain insight into the outcomes of patients with and without a delayed diagnosis.

We could not study the clinical consequences of diagnostic delay since only a few of the included studies reported on clinical outcomes, such as recurrent PE or mortality. For instance, none of the included studies reported on clinical outcomes such as chronic thromboembolic pulmonary hypertension (CTEPH) or post-embolic syndrome. However, we know from the sparsely existing literature on post-embolic syndromes that a delayed diagnosis might be a risk factor for developing CTEPH [38].

## Conclusion

Delay in diagnosing PE is common. Patients may have symptoms for almost one week before PE is diagnosed; in about a quarter of patients the diagnostic delay is even longer.

## Supplementary Material

Appendix 5: PRISMA Checklist

Appendix 4: Factors associated with diagnostic delay

Appendix 3: Risk of bias & applicability

Appendix 2: Risk of bias & applicability (based on QUADAS-2 tool)

Appendix 1: Search: review diagnostic delay pulmonary embolism
