# Supplementary material for: A systematic review and meta-analysis of diagnostic delay in pulmonary embolism
Source: Eur J Gen Pract. 2022 Jun 22;28(1):165–72. doi: 10.1080/13814788.2022.2086232 (PMC9246192; doi:10.1080/13814788.2022.2086232)
Supplement: Appendix 4: Factors associated with diagnostic delay [file IGEN_A_2086232_SM7436.docx]

**Appendix 4. Factors associated with diagnostic delay**

|  | | | **Patient characteristics** | | **Symptoms** | | | | | **Comorbidity** | | | | **Risk factors** | | | | | **Physical**  **examination** | |
| --- | --- | --- | --- | --- | --- | --- | --- | --- | --- | --- | --- | --- | --- | --- | --- | --- | --- | --- | --- | --- |
|  |  |  | Gender  (male) | Age | Dyspnoea | Chest pain | Cough | DVT  (symptoms) | Syncope | Cancer | Chronic  lung  disease | Heart  Failure | Prior  Pulmonary  Infection | Previous  VTE | Smoking | Immobilization | Recent surgery | Oestrogen use | Hypotension | Tachycardia |
|  | | | *Univariable analysis* | | | | | | | | | | | | | | | | |  |
| Ageno | | 2008 | **= (-)** | **= (-)** | **= (+)** | **= (-)** |  |  | **= (-)** | **= (-)** |  |  |  | **= (-)** |  |  |  |  |  |  |
| Alonso-Mar. | | 2010 |  | **+** |  | **= (-)** |  |  | **-** |  |  |  |  | **-** |  |  |  |  |  |  |
| Bulbul | | 2009 | **= (+)** | **= (-)** | **= (-)** | **= (-)** | **+** | **= (-)** |  |  |  |  |  |  |  |  |  |  | **-** | **= (-)** |
| Bulbul | | 2011 |  | **= (+)** | **= (+)** | **= (+)** |  | **= (+)** | **-** |  |  |  |  |  | **= (+)** |  | **-** |  | **-** |  |
| Den Exter | | 2013 | **= (+)** | **+** |  |  |  |  |  | **= (-)** | **+** | **+** |  | **= (+)** |  | **-** | **-** | **-** |  |  |
| Goyard | | 2018 | **= (-)** | **= (+)** | **+** | **= (-)** |  | **= (+)** | **-** | **= (-)** | **= (+)** | **= (+)** |  | **-** |  | **-** | **-** | **= (+)** | **= (-)** | **= (-)** |
| Hendriksen | | 2017 | **= (-)** | **+** | **= (+)** | **-** | **+** | **-** | **= (-)** | **= (+)** | **+** | **= (+)** | **+** | **= (+)** | **= (-)** | **= (-)** | **= (-)** | **= (-)** |  | **= (-)** |
| Jenab | | 2014 | **= (+)** | **= (+)** | **+** | **-** |  | **+** | **= (-)** | **= (+)** |  | **= (+)** |  | **= (-)** | **= (+)** | **-** | **-** | **-** | **= (-)** | **= (-)** |
| Jimenez | | 2007 | **= (+)** | **(-)** | **= (+)** | **= (+)** |  | **= (+)** | **+** | **= (+)** | **= (+)** | **= (+)** |  | **= (+)** |  | **= (-)** | **-** |  | **= (-)** | **= (-)** |
| Ozsu | | 2011 |  | **= (+)** |  |  |  | **= (+)** | **-** | **= (-)** |  | **= (+)** |  | **= (-)** |  | **+** | **-** | **= (+)** | **= (-)** | **= (+)** |
| Pasha | | 2014 | **= (-)** | **= (+)** |  |  |  |  |  | **= (-)** | **= (+)** | **= (+)** |  | **= (-)** |  | **= (+)** |  |  |  |  |
| Walen | | 2016 | **= (+)** | **= (+)** | **+** | **-** | **= (+)** | **= (-)** |  | **= (-)** |  |  |  | **= (+)** |  |  |  |  |  | **= (-)** |
|  | | | *Multivariable analysis* | | | | | | | | | | | | | | | | |  |
| Bulbul | 2009 | |  |  |  | **= (-)** | **+** |  |  |  |  |  |  |  |  |  |  |  | **-** |  |
| Chan | 2020 | |  | **+** | **-** |  |  |  | **+** |  | **+** | **+** |  |  |  |  |  |  |  |  |
| Den Exter | 2013 | |  | **+** |  |  |  |  |  |  | **+** |  |  |  |  | **-** | **-** |  |  |  |
| Goyard | 2018 | | **= (-)** | **= (+)** | **+** | **= (-)** |  | **= (+)** | **-** |  | **= (+)** |  |  | **-** |  | **-** | **-** | **= (-)** | **= (-)** | **= (-)** |
| Hendriksen | 2017 | | **-** | **+** | **-** | **-** |  |  |  |  | **+** |  | **+** |  |  |  |  |  |  |  |
| Kayhan | 2012 | |  | **= (-)** |  |  |  |  |  |  |  |  |  |  | **-** |  |  |  |  |  |
| Ozsu | 2011 | |  |  |  |  |  |  | **= (-)** | **-** |  |  |  | **= (-)** |  | **= (+)** | **-** | **= (+)** |  |  |
| Walen | 2016 | |  |  | **= (+)** | **-** | **= (+)** | **-** |  |  |  |  |  |  |  |  |  |  |  |  |

*+ Positive association with delay*

*- Negative association with delay*

*= No statistically significant association with delay*
