# Supplementary material for: A systematic review and meta-analysis of diagnostic delay in pulmonary embolism
Source: Eur J Gen Pract. 2022 Jun 22;28(1):165–72. doi: 10.1080/13814788.2022.2086232 (PMC9246192; doi:10.1080/13814788.2022.2086232)
Supplement: Appendix 3: Risk of bias & applicability [file IGEN_A_2086232_SM7435.docx]

**Appendix 3. Risk of bias & Applicability**

|  | Risk of bias | | |  | Applicability to  primary care |
| --- | --- | --- | --- | --- | --- |
| Study | Patient selection | Valid diagnosis | Assessment of delay |  |  |
| Ageno 2008 | Low | Low | Low |  | Likely |
| Alonso-Martinez 2004 | Low | Low | Unclear |  | Not |
| Alonso-Martinez 2010 | Low | Low | Unclear |  | Not |
| Aranda 2021 | High | Low | Unclear |  | Not |
| Aydogdu 2013 | Low | Low | Unclear |  | Possibly |
| Berghaus 2011 | Low | Low | High |  | Not |
| Bulbul 2009 | Low | Low | High |  | Unclear |
| Bulbul 2011 | High | Low | Low |  | Unclear |
| Chan 2020 | Low | Low | High |  | Not |
| Den Exter 2013 | Low | Low | Unclear |  | Likely |
| Elliott 2005 | Unclear | Low | Unclear |  | Unclear |
| Goyard 2018 | Low | Low | Low |  | Unclear |
| Hendriksen 2017 | Low | Low | High |  | Very |
| Ilvan 2015 | Low | Low | High |  | Possibly |
| Jenab 2014 | Low | Low | Low |  | Possibly |
| Jimenez 2007 | Low | Low | Unclear |  | Possibly |
| Kayhan 2012 | Low | Low | High |  | Not |
| Menéndez 1998 | High | Low | High |  | Unclear |
| Ozlem 2016 | High | Low | High |  | Possibly |
| Ozsu 2011 | High | Low | High |  | Likely |
| Pasha 2014 | Low | Low | Low |  | Likely |
| Rahimi-Rad 2013 | Low | Low | Unclear |  | Unclear |
| Walen 2016 | High | Low | High |  | Very |
| Zycinska 2013 | Low | Unclear | Unclear |  | Likely |
