# Supplementary material for: A systematic review and meta-analysis of diagnostic delay in pulmonary embolism
Source: Eur J Gen Pract. 2022 Jun 22;28(1):165–72. doi: 10.1080/13814788.2022.2086232 (PMC9246192; doi:10.1080/13814788.2022.2086232)
Supplement: Appendix 2: Risk of bias & applicability (based on QUADAS-2 tool) [file IGEN_A_2086232_SM7434.docx]

**Appendix 2.** Risk of bias & applicability (based on QUADAS-2 tool)

| **Risk of bias assessment** | | | | | | |
| --- | --- | --- | --- | --- | --- | --- |
| **Domain** | **Patient selection** | | **Valid diagnosis** | | **Assessment of delay** | |
| **Description** | Describe methods of patient selection: Describe included patients? | | Describe the test used for final diagnosis. | | Describe the method of assessment of delay. | |
| **Signalling questions** | Was a consecutive or random sample of patients enrolled? | | Was a CT-scan, V/Q-scan, perfusion scan or ultrasound proven DVT with PE symptoms performed? | | - What was the study type?  - Risk of recall bias?  - Was the delay reported by patients/ doctor/ both?  - Was the health record of the patient used? | |
|  | Did the study avoid inappropriate exclusions?* | |  | |  | |
| **Risk of bias: High/low/unclear** | Could the selection of patients have introduced bias? | | Could the test used for diagnosis have introduced bias? | | Could the assessment of delay have introduced bias? | |
| **Applicability to primary care** | | | | | | |
| **Signalling question** | Are the included patients in the original studies comparable to patients in primary care? | | | | | |
| Very applicable: Patients included in primary care OR Patients referred by a general practitioner | Likely applicable:  Patients partly included in primary care or outpatient clinic | Possibly applicable:  Patients included in emergency departments | | Not applicable:  Patients included in hospital wards during admission | | Unclear:  Not clearly explained where and how patients are included |

* >5% exclusion due to lost to follow-up was classified as ‘high’ risk of bias
