# Supplementary material for: A systematic review and meta-analysis of diagnostic delay in pulmonary embolism
Source: Eur J Gen Pract. 2022 Jun 22;28(1):165–72. doi: 10.1080/13814788.2022.2086232 (PMC9246192; doi:10.1080/13814788.2022.2086232)
Supplement: Appendix 1: Search: review diagnostic delay pulmonary embolism [file IGEN_A_2086232_SM7432.docx]

**Appendix 1. Search: review diagnostic delay pulmonary embolism**

**Pubmed**

(((((((Pulmonary Embolism*[tiab]) OR (Pulmonary Infarct* [tiab]) OR (Pulmonary Embolism[Mesh]) OR (pulmonary thromboembolism* [tiab]))))) OR "Venous Thromboembolism"[Mesh])) OR ((lung embol*[Title/Abstract] OR lung infarct*[Title/Abstract]))

AND

((((diagnos*[Title/Abstract]) AND (late[Title/Abstract] OR delay*[Title/Abstract] OR missed[Title/Abstract] OR missing[Title/Abstract] OR error*[Title/Abstract] OR inappropriate*[Title/Abstract] OR time[Title/Abstract] OR timing[Title/Abstract] OR timely[Title/Abstract]))) OR (("Delayed Diagnosis"[Mesh]) OR "Diagnostic Errors"[Mesh])) OR (misdiagnos*[tiab] OR undiagnos*[tiab])

**Embase**

‘pulmonary embolism*’:ti,ab,kw OR ‘pulmonary infarct*‘:ti,ab,kw OR ‘lung embolism*‘:ti,ab,kw OR ‘pulmonary thromboembolism*‘:ti,ab,kw OR ‘venous thromboembolism*‘:ti,ab,kw OR ‘lung infarction*‘:ti,ab,kw OR ‘lung embolism‘/exp

AND

(‘diagnos*’:ti,ab,kw AND (‘late’:ti,ab,kw OR ‘delay’:ti,ab,kw OR ‘missing’:ti,ab,kw OR ‘missed’:ti,ab,kw OR ‘error’:ti,ab,kw OR ‘inappropiate*’:ti,ab,kw OR ‘time’:ti,ab,kw OR ‘timely’:ti,ab,kw OR ‘timing’:ti,ab,kw)) OR (‘delayed diagnos*’:ti,ab,kw OR ‘delayed diagnosis’/exp OR ‘diagnostic error*’:ti,ab,kw OR ‘diagnostic error’/exp OR ‘misdiagnos*’:ti,ab,kw OR ‘undiagnos*’:ti,ab,kw)
